# mHealth Interventions to Reduce Physical Inactivity and Sedentary Behavior in Children and Adolescents: Systematic Review and Meta-analysis of Randomized Controlled Trials

**DOI:** 10.2196/35920

**Published:** 2022-05-11

**Authors:** Hannes Baumann, Janis Fiedler, Kathrin Wunsch, Alexander Woll, Bettina Wollesen

**Affiliations:** 1 Department of Human Movement Faculty of Psychology and Human Movement Science University of Hamburg Hamburg Germany; 2 Department of Biological Psychology and Neuroergonomics Institute for Psychology and Occupational Science Technical University Berlin Berlin Germany; 3 Department of Performance, Neuroscience, Therapy and Health Faculty of Health Sciences Medical School Hamburg Hamburg Germany; 4 Institute of Sports and Sports Science Karlsruhe Institute of Technology Karlsruhe Germany

**Keywords:** health behavior change, individualization, sedentary behavior, physical activity, tailored interventions, personalized medicine, health app, mobile phone

## Abstract

**Background:**

Children and adolescents increasingly do not meet physical activity (PA) recommendations. Hence, insufficient PA (IPA) and sedentary behavior (SB) among children and adolescents are relevant behavior change domains for using individualized mobile health (mHealth) interventions.

**Objective:**

This review and meta-analysis investigated the effectiveness of mHealth interventions on IPA and SB, with a special focus on the age and level of individualization.

**Methods:**

PubMed, Scopus, Web of Science, SPORTDiscus, and Cochrane Library were searched for randomized controlled trials published between January 2000 and March 2021. mHealth interventions for primary prevention in children and adolescents addressing behavior change related to IPA and SB were included. Included studies were compared for content characteristics and methodological quality and summarized narratively. In addition, a meta-analysis with a subsequent exploratory meta-regression examining the moderating effects of age and individualization on overall effectiveness was performed.

**Results:**

On the basis of the inclusion criteria, 1.3% (11/828) of the preliminary identified studies were included in the qualitative synthesis, and 1.2% (10/828) were included in the meta-analysis. Trials included a total of 1515 participants (mean age (11.69, SD 0.788 years; 65% male and 35% female) self-reported (3/11, 27%) or device-measured (8/11, 73%) health data on the duration of SB and IPA for an average of 9.3 (SD 5.6) weeks. Studies with high levels of individualization significantly decreased insufficient PA levels (Cohen *d*=0.33; 95% CI 0.08-0.58; *Z*=2.55; *P*=.01), whereas those with low levels of individualization (Cohen *d*=−0.06; 95% CI −0.32 to 0.20; *Z*=0.48; *P*=.63) or targeting SB (Cohen *d*=−0.11; 95% CI −0.01 to 0.23; *Z*=1.73; *P*=.08) indicated no overall significant effect. The heterogeneity of the studies was moderate to low, and significant subgroup differences were found between trials with high and low levels of individualization (*χ*^2^_1_=4.0; *P*=.04; *I*^2^=75.2%). Age as a moderator variable showed a small effect; however, the results were not significant, which might have been because of being underpowered.

**Conclusions:**

Evidence suggests that mHealth interventions for children and adolescents can foster moderate reductions in IPA but not SB. Moreover, individualized mHealth interventions to reduce IPA seem to be more effective for adolescents than for children. Although, to date, only a few mHealth studies have addressed inactive and sedentary young people, and their quality of evidence is moderate, these findings indicate the relevance of individualization on the one hand and the difficulties in reducing SB using mHealth interventions on the other.

**Trial Registration:**

PROSPERO CRD42020209417; https://www.crd.york.ac.uk/prospero/display_record.php?RecordID=209417

## Introduction

### Rationale

“Inactivity is the epidemic of the 21st century” [1]. The prevalence of insufficient physical activity (IPA; defined as not meeting the specified physical activity [PA] guidelines [2]) in children and adolescents is >80% worldwide, which is mainly attributable to time spent on sedentary behavior (SB; defined as any waking behavior characterized by an energy expenditure ≤1.5 metabolic equivalents of task [METs] while in a sitting, reclining, or lying posture [2,3]) and has increased continuously over the past decades [4]. This trend remains unbroken, although the health benefits of at least 60 minutes of moderate to vigorous PA (MVPA; defined as any activity with a MET value between 3 and 5.9; vigorous-intensity PA is defined as ≥6 METs [5,6]) on average per day for children and adolescents are well-established [7].

Although SB and IPA may be used synonymously, and indeed by definition, they refer to the same energy expenditure spectrum, it should still be noted that they are not necessarily correlated [8], and both have severe health consequences [9]. For example, children and adolescents may exhibit high levels of SB (driving to school, sitting in class all day, and playing video games in the evening) while simultaneously meeting the recommended PA guidelines (going to soccer practice for an hour in the evening). In this case, the health consequences of SB time would be occurring, although the PA level is sufficient. If IPA and SB are performed in childhood and adolescence, it is assumed that these behavioral patterns will endure until adulthood [10], which is why, from a global perspective, it is important to target young populations with strong IPA and SB patterns in the context of primary prevention.

Given the increasing digitization in health care and the proliferation of smartphones [11], mobile health (mHealth) interventions have been shown to be effective and of scope in reducing IPA and SB in children and adolescents [12], as well as in adults [13]. A more detailed glance at the contents of mHealth interventions reveals that SMS text messaging interventions are one of the most common methods used for delivering mHealth interventions [14], which has been recently criticized [15]. Instead, personalized approaches should focus on responding appropriately to the realities of everyday life and addressing the diversity of modern societies [16]. Key facets of effective mHealth interventions depict the integration of behavior change techniques (BCTs) [17] and the foundation upon existing theoretical approaches [18]. Furthermore, there is empirical evidence that just-in-time interventions [19,20], individualized or tailored interventions [21], and interventions that incorporate multiple BCTs [22] show large potential in this respect. However, Chen et al [23] highlight that the design of mHealth interventions often lacks a theory-driven approach [24,25], and there is little emphasis on evidence-based content [26]. Another difficulty with mHealth interventions occurs when existing evidence is summarized in meta-analyses and refers to outcomes that are coreported as secondary outcomes but do not constitute the core of the intervention [27].

Until recently, there have been far more mHealth interventions for healthy adults aiming to reduce IPA and SB than for healthy children and adolescents [13,28]. In one of the very few reviews on healthy children and adolescent target groups, Schoeppe et al [12] demonstrated an overall moderate quality of health apps and found a positive correlation between app quality and the number of app features and BCTs, therefore suggesting that future apps should target user engagement, be tailored to specific populations, and be guided by health behavior theories. Böhm et al [28] furthermore criticize the quality of mHealth interventions for children and adolescents in this respect and suggest that more age-appropriate solutions are needed. The results of other reviews indicate that smartphone-based mHealth interventions (especially apps) are a versatile strategy for increasing PA and steps in children and adolescents [29]. For example, Laranjo et al [30] found an average increase of 1850 steps per day after an mHealth intervention. However, it is also occasionally mentioned that the use of mHealth could lead to a further increase in the already high screen time of children and adolescents [31,32], which needs to be taken into consideration when planning and implementing mHealth apps. Although mHealth can increase screen time, it may not necessarily do so. The representative and longitudinal Motorik-Modul study demonstrated that increased screen time does not correlate with PA minutes, opening various opportunities for digital interventions and potential ways for new approaches to target the IPA and SB of children and adolescents [33,34].

In the context of mHealth, individualization is defined as an adaptation to the needs or special circumstances of an individual and is cited as one of the main barriers that prevent patients from changing their health behavior [23,35]. Individualized interventions (sometimes also called adaptive, needs-specific, target group–specific, tailored, or personalized interventions) offer a potential way of delivering person-centered interventions by varying levels of individual needs and empowering individuals to monitor their health actively [21]. Non-mHealth interventions have sometimes used individualized one-on-one meetings, showing high effectiveness but consuming much time and resources. Therefore, this approach has been criticized as time consuming and resource burdening [36,37]. Apps can apply this approach in a much more ecological way by being easily accessible to a wide variety of populations. The enhanced efficacy of individualized interventions compared with nonindividualized interventions has been repeatedly demonstrated in various populations [30,38,39], especially in adults [40], but not yet in children or adolescents, although several randomized controlled trials address this matter. For example, the MOPO study examined the effects of a gamified and individualized mHealth intervention and has not been cited in any meta-analysis to date [41]. Another example of this is the intervention of Moreau et al [42], which is a fully automated, theory-driven, tailored intervention. In addition, there is no existing taxonomy for individualized app elements as there is, for example, for behavior change mechanisms [17], from which derives the urgent need for further systematic reviews and development of a taxonomy for individualized elements.

### Objective

Although several reviews [12,28,29,43] have been published on mHealth-based PA promotion in children and adolescents, and some of them also include studies with IPA and SB as outcomes, none of the existing reviews ensures (1) a clear focus on the at-risk target group of children and adolescents with high IPA and SB levels and (2) a separate analysis of effects of mHealth on IPA and SB. Therefore, this review might contribute to a better understanding of the needs of children and adolescents who engage in IPA and high SB. For this reason, this review’s aims were 3-fold.

First, there is a need to identify and describe existing SB and IPA mHealth interventions that address PA for children and adolescents. Second, this review sought to answer whether and how mHealth interventions are effective in reducing IPA and SB in healthy children and adolescents. Third, there is a need to explore whether age and individualization are moderators of the overall effectiveness of the mHealth interventions. This leads to the following main research questions:

1. What are the characteristics of effective existing mHealth interventions for children and adolescents to reduce SB and IPA?
2. How effective are existing mHealth interventions for children and adolescents in reducing SB and IPA?
3. What moderating effects do individualization and age have on the effectiveness of mHealth interventions for children and adolescents to reduce SB and IPA?

## Methods

This systematic review and meta-analysis was conducted according to Cochrane methodology, and the results were reported following the PRISMA (Preferred Reporting Items for Systematic Reviews and Meta-Analyses) 2020 statement [44].

### Eligibility Criteria

The criteria for eligible studies are defined in accordance with the population, intervention, comparison, and outcomes criteria [45] and are presented in Textbox 1. In line with World Health Organization (WHO) recommendations [5], IPA was defined as <60 minutes of MVPA per day or insufficient step count per day (<5000 steps per day) [46], and SB was defined as any waking behavior characterized by an energy expenditure of ≤1.5 METs while in a sitting, reclining, or lying posture [2,3]. Alternative measures can be screen time and sitting time.

**Summary of the population, intervention, comparison, and outcomes and eligibility criteria.**

**Participants and population**

- Inclusion: healthy children and adolescents (aged 0-21 years) without physical or psychological morbidities that would influence the realization of behaviors targeted by the respective interventions and studies that include participants with any physical or psychological morbidities (eg, populations with obesity) and provides a subgroup analysis for the healthy population separately
- Exclusion: children and adolescents with any physical or psychological morbidities, populations with mean age >21 years, studies conducted within clinical settings, and studies focusing on populations whose insufficient physical activity (IPA) or sedentary behavior (SB) is influenced by disease-specific recommendations or health status

**Intervention or interventions and exposure or exposures**

- Inclusion: mobile health (mHealth) interventions with healthy children and adolescents where the primary or secondary outcome measure was IPA or SB, mixed interventions, and family-based interventions
- Exclusion: studies without mHealth interventions

**Comparator(s) and control**

- Inclusion: active or passive control groups
- Exclusion: studies without a control group

**Outcomes**

- Inclusion:
  - IPA, which is defined as <60 minutes of self-reported or accelerometry-measured moderate to vigorous physical activity per day or insufficient step count per day (<5000 steps per day); therefore, various physical activity measures (min/week of physical activity, steps, counts, metabolic equivalents of task [MET] minutes, screen time, and sitting time) need to be included
  - SB, which is defined as any waking behaviors characterized by an energy expenditure of ≤1.5 METs while in a sitting, reclining, or lying posture; alternative measures can be screen time and sitting time
- Exclusion: mHealth intervention studies that do not involve IPA or SB as a primary or secondary outcome

**Types of study to be included**

- Inclusion: randomized controlled trials (RCTs) that include individual or cluster randomization, clinical trials, feasibility studies with an RCT design, and just-in-time adaptive interventions; for a potential meta-analysis, only RCTs were included
- Exclusion: nonexperimental study designs (eg, observational or case studies, studies reporting prevalence or trend data, measurement studies, and theoretical papers), non–peer-reviewed studies, and nonprimary studies (eg, letters, comments, conference proceedings, reviews, and narrative articles)

### Information Sources

After group discussion among the research team, a systematic search for randomized controlled trials in English between January 1, 2000, and January 29, 2021. was conducted using the 5 databases of PubMed, Scopus, Web of Science, SPORTDiscus, and Cochrane Library.

### Search Strategy

The search terms were reviewed by 3 authors (HB, JF, and KW), and the search was conducted by 1 author (HB) in March 2021. The following vital constructs, as well as numerous synonyms, were used: (*children* OR *adolescents*) AND (*mHealth*) AND (*IPA* OR *SB*). The entire search strategy can be found in the *Availability of Data, Code, and Other Materials* section.

### Selection Process

**Figure 1:**
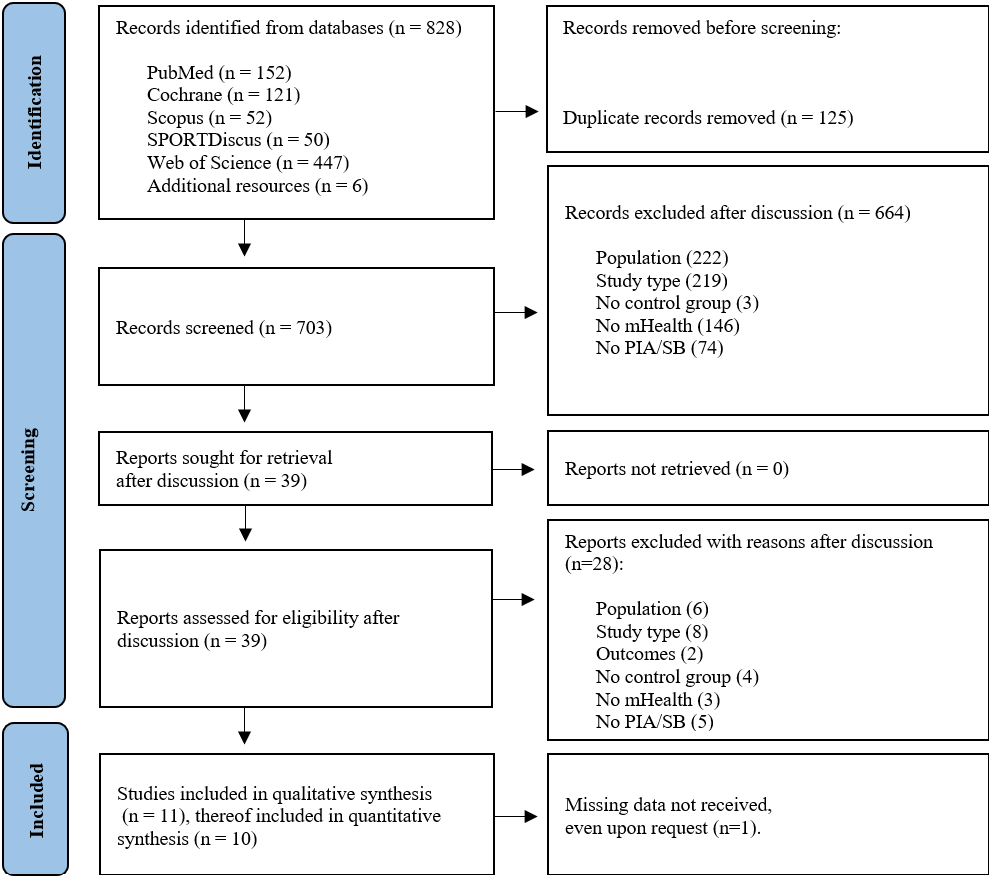
Flowchart and study selection process (adapted from Page et al [45]). IPA: insufficient physical activity; SB: sedentary behavior.

The identified literature was imported to the reference management software Zotero (Roy Rosenzweig Center for History and New Media). After removing duplicates, the first author (HB) and a coauthor (JF) screened titles and abstracts to identify all potentially eligible studies based on the inclusion and exclusion criteria (the detailed study flow is presented in the PRISMA flowchart in Figure 1). Full-text articles were retrieved for eligible abstracts and reviewed by the same 2 authors before inclusion in the review. The first author (HB) and a second reviewer (JF) independently assessed full paper copies of remaining potentially eligible studies to determine included studies, and if no consent was reached, a third reviewer (KW) resolved the disagreement by discussion and arbitration.

### Data Collection Process and Data Items

On a study level, data, including the name of the author, year of publication, study type, study aim, information about the mHealth intervention, duration of intervention, follow-up period, target population or setting, integration of parents, country, sample size, age (range, mean, and SD), gender, IPA or SB criterion, relevant outcomes, measurement method, treatment effects, individualized elements, BCT elements, and theoretical foundation were extracted. To identify interventions with high and low levels of individualization, we quantified the individualized elements and defined *low level of individualization* as the number of individualized items below the IQR of the evaluated interventions and *high level of individualization* as the number of individualized items within or above the IQR of the evaluated interventions.

### Study Risk of Bias Assessment

The risk of bias (ROB) in individual studies was evaluated independently by 2 reviewers (HB and KW) using the 5-dimensional ROB 2 tool [47]. In this procedure, the overall ROB is classified as low if all dimensions indicate low risk. Once ≥1 dimension is rated as unclear, the entire trial is rated the same way. Furthermore, if ≥1 dimension is classified as being high risk, the overall ROB is rated high. Disagreements between the authors concerning the ROB were resolved by discussion, with the involvement of another author where necessary.

### Effect Measures

To perform a meta-analysis, the sample sizes, means, and SDs of measurement time points 1 and 2 were extracted from the intervention and control groups of all included studies (or study arms) for both IPA and SB. For reasons of comparability in the meta-analysis, follow-up data were not extracted, as not all studies included a third or fourth measurement point. When multiple primary outcome measures were presented, the most conclusive measure to our research questions was identified by JF and HB. Quality of information and the orientation toward WHO guidelines played a critical role in this process. It was defined that IPA was most likely to be modeled by *minutes of MVPA per day*, as suggested by the WHO, followed by *minutes of light MVPA per day*, *minutes of PA per day*, and *number of steps per day*. For SB, *minutes in SB per day* was preferred over the proxy measures of *minutes of sitting time per day* and *minutes of screen time per day*.

### Synthesis Methods

If data for the meta-analysis were not available in the original manuscripts, the study authors were contacted. The last search was conducted in March 2021. Extracted data were then weighted by sample size (splitted shared group procedure was used in studies with multiple study arms to avoid unit of analysis error [48]), converted into Cohen *d*, and integrated into a meta-analysis with random effects using RevmanWeb [49] calculator. We used the following benchmark to interpret the effect sizes: effect sizes >0.50 are interpreted as large, effect sizes of 0.50 to 0.30 as moderate, and effect sizes of 0.30 to 0.10 as small or <0.10 as trivial [50]. Tests for heterogeneity, overall effects, and subgroup differences were also calculated using RevmanWeb.

### Reporting Bias Assessment and Certainty Assessment

To assess publication bias, funnel plots were compiled using RevmanWeb to determine asymmetric shapes within the natural statistical dispersion [51]. If the plot is asymmetric because of many large effect sizes on one side of the mean, it strongly suggests unpublished or unconducted studies with contrary results. To provide certainty of the evidence, the Grading of Recommendations, Assessment, Development, and Evaluations approach [52] was used as an extension of the ROB assessment. The following five factors were examined to obtain a well-founded assessment: individual study limitations (ROB), inconsistency of results (heterogeneity), indirectness of evidence (external validity), imprecision (small sample size and wide CI), and publication bias.

### Additional Analyses

An additional meta-regression was performed in R-Studio [53] using the *Metafor* package [54] to relate the estimated effect sizes to the mean age of the samples. We distinguished between primary outcome (IPA or SB) and level of individualization (low or high). The included trials (and their multiple arms) were divided into trials with high (number of individualized items within or above the IQR of evaluated interventions) and low levels of individualization (number of individualized items below the IQR of evaluated interventions) to conduct a meta-analysis. For both IPA and SB outcomes, a separate meta-analysis was conducted to provide the comparability of effects. To visualize the results, a grouped bubble plot was created in Microsoft Excel [55], plotting the weighted standardized mean differences of the individual trials and the average age of the participants. Group differentiation was based on the primary outcome (IPA and SB).

### Registration and Protocol

The protocol for this systematic review and meta-analysis was prospectively registered on PROSPERO (International Prospective Register of Systematic Reviews) and can be accessed using registration number CRD42020209417.

### Availability of Data, Code, and Other Materials

The search string (Medical Subject Headings) was as follows:

(Child [MeSH] OR Adolescent [MeSH]) AND (Health Promotion[MeSH] OR School Health Services[MeSH] OR Primary Prevention[MeSH] OR Health Behavior Change) AND (Telemedicine [MeSH] OR Patient-Specific Modeling[MeSH] OR Individuali* OR tailored Intervention OR digital health OR Mobile Applications[MeSH] OR mobile phone* OR smartphone* OR iPhone* OR iPad* OR tablet* OR android OR SMS OR text message* OR App OR Reminder Systems [MeSH]) AND (SB[MeSH] OR Physical Fitness[MESH] OR Exercise[MESH] OR energy expenditure) / Filter applied: years 2010-2020, only RCT and Clinical Trials

## Results

### Study Selection

The initial database search generated 828 articles, of which 125 (15.1%) were duplicates (Figure 1), and the study screening identified 11 (1.35) studies as eligible for qualitative analysis and 10 (1.2%) articles for quantitative synthesis.

### Study Characteristics

A total of 11 randomized controlled trials were included (n=10, 91%, parallel and n=1, 9%, crossover trial), with a duration of 9.3 (SD 5.6) weeks, of which 3 (27%) [56-58] included a follow-up measurement. Eligible trials included samples of 40 to 496 participants (mean 138, SD 145), with a mean age range of 3.5 to 17.8 years (Table 1). In 9% (10/11) of studies, both genders were approximately equally represented. A single study [41] only included male adolescents, resulting in an overall gender distribution of 975 boys and young men to 540 girls and young women. Approximately 27% (3/11) of trials with young children (aged <5 years) included parent integration, whereas others focused on children and adolescents only. The target population and study aims varied across studies, and the countries were exclusively Western nations. The mHealth interventions ranged from basic SMS text messaging interventions to web-based mobile interventions, individualized and gamified apps, and wearable interventions. In addition, of the 15 interventions, 3 (20%) used self-reported measures, and 8 (53%) interventions used device-based measures of health data on the duration of SB and IPA. Furthermore, it should be mentioned that not all studies focused on reducing SB or IPA as their primary objective. Approximately 45% (5/11) of studies aimed to promote PA [41,57-60], 9% (1/11) aimed to improve fat mass index [61], 9% (1/11) aimed to reduce BMI [62], and 9% (1/11) aimed to change behavior [56] as a primary study aim.

**Figure 2:**
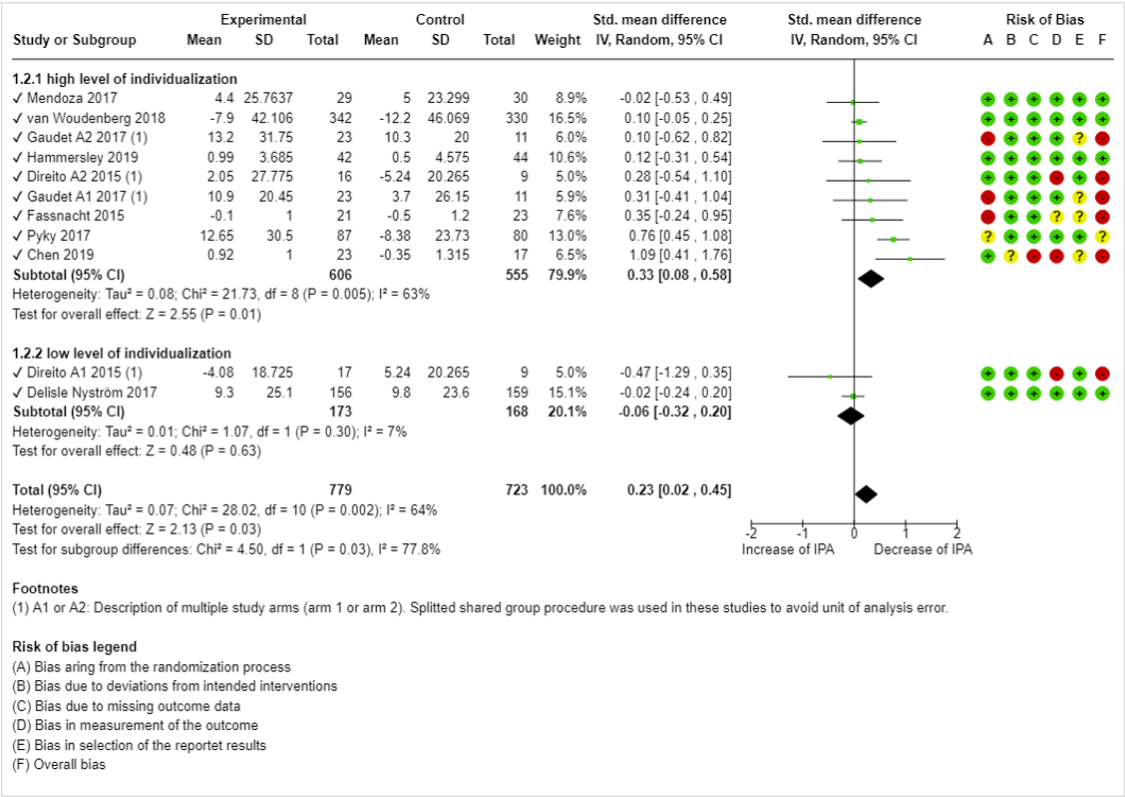
Forest plot for effect size comparison of high-individualized versus low-individualized mobile health interventions on decreasing IPA [42,58-63,66]. IPA: insufficient physical activity.

**Figure 3:**
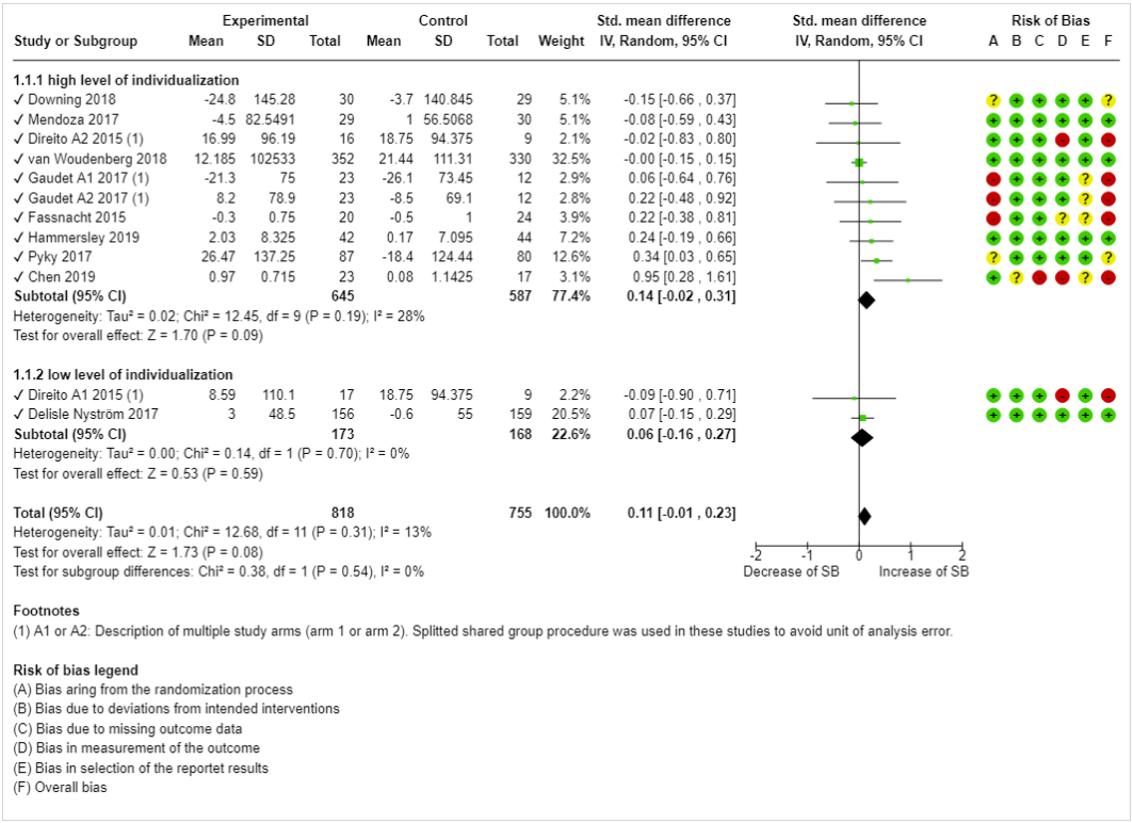
Forest plot for effect size comparison of high-individualized versus low-individualized mobile health interventions on decreasing SB [42,58-64,66]. RCT: randomized controlled trial; SB: sedentary behavior.

The quantitative results of the individual studies are presented in the forest plots in Figures 2 and 3. To describe each intervention (or study arm) in detail, the number and content of individualized elements, BCTs, and theoretical foundations are presented in Table 2.

**Table 1:** Study characteristics.

Study	Study type (duration in weeks)	Study aim	Description of mHealth^a^ intervention	Population (setting), region, and country	Sample size (N)	Age (years)			SB^b^ (unit) and IPA^c^ outcomes (unit); measurement method
						Values, mean (SD)	Values, range		
Chen et al [62]	2-arm parallel RCT^d^ with follow-up (12)	Decrease BMI	iStart Smart for Teens: a smartphone-based, culturally appropriate, and tailored educational program for weight management	Chinese American adolescents who are overweight, California, United States	40 (male 23 and female 17)	14.9 (1.67)	13-18	SB (hours per day) and PA^e^ (days per week); questionnaire (California Health Interview Survey)	
Nyström et al [61]	2-arm parallel RCT (24)	Reduce obesity (improve fat mass index)	Web-based app to deliver MINISTOP intervention, which provided an extensive program of information and behavioral support	Healthy children (preschool; parental support), Östergötland, Sweden	313 (male 170 and female 143)	4.5 (0.1)	4-5	SB (min/day) and MVPA^f^ (minutes per day); ActiGraph wGT3x-BT accelerometer	
Direito et al [58]	3-arm parallel RCT (8)	Improve PA levels in healthy young people who are insufficiently active	AIMFIT trial compared the apps “Zombies, run” and “Get Running” with a control group (device measured)	Healthy adolescents, Auckland, New Zealand	51 (male 22 and female 29)	15.67 (1.2)	14-17	SB (minutes per day) and MVPA (minutes per day); accelerometer (ActiGraph GT1M) and PAQ-A^g^	
Downing et al [63]	2-arm pilot RCT (6)	Reducing children’s SB in early age	Mini-Movers: SMS text messaging intervention to provide information and practical support	Young children (playgroups; parental support), Melbourne, Australia	57 (male 26 and female 31)	3.05 (0.75)	2-4	Sitting time (minutes per day) and no IPA outcome; ActivePAL	
Fassnacht et al [59]	2-arm parallel RCT (8) with 2 follow-ups (4 and 4)	Promote health behavior in school-aged children	Daily behavior reporting and feedback vis SMS text messaging	Healthy children (elementary school), Braga, Portugal	49 (male 23 and female 26)	9.6 (0.4)	8-19	Screen time (hours per day) and PA (hours per day); Family Eating and Activity Habits questionnaire	
Gaudet et al [57]	Crossover RCT (6)	Increase PA in young adolescents	Wrist-worn PA tracker (Fitbit, model Charge HR)+web-based Fitbit user account	Young adolescents (school), New Brunswick, Canada	46, (male 22 and female 24)	13.0 (0.35)	13-14	SB (minutes per day) and MVPA (minutes per day); Actical accelerometer	
Hammersley et al [64]	2-arm parallel RCT (11) with 2 follow-ups (12 and 24)	Reduce obesity behaviors in preschool children	Parent focused; Time2bHealthy Online Program with Fakebook integration	Children who are overweight (preschool; parental support), Wollongong, Australia	86 (male 43 and female 43)	3.46 (0.92)	2-5	SB (minutes per day) and MVPA (minutes per day); ActiGraph GT3X+ accelerometer	
Mendoza et al [60]	2-arm parallel RCT (10)	Promote PA among adolescent and young adult survivors	Wearable PA-tracking device (Fitbit Flex) and a peer-based web-based support group (a Facebook group)	Childhood survivors of cancer, Seattle, United States	59 (male 24 and female 35)	16.6 (1.5)	14-18	SB (minutes per day) and MVPA (minutes per day); ActiGraph GT3X+ accelerometer	
Pyky et al [41]	2-arm parallel RCT (6)	Promote PA and social activity	Game-based persuasion, for example, by physically moving within the districts of the city; players could earn points and claim areas for their clan in-game	Young adolescent men (military), Oulu, Finland	496 (male 496 and female 0)	17.8 (0.6)	16-20	SB (minutes per day) and MVPA (minutes per day); Polar Active Accelerometer	
Sirriyeh et al [56]	4-arm exploratory RCT (2)	PA behavior change	Daily SMS text messages, which included manipulations of affective or beneficial beliefs	Late adolescents (state schools), Yorkshire, United Kingdom	128 (male 38 and female 90)	17.3 (0.68)	16-19	IPAQ^h^ questionnaire; no outcomes; time point 0 data missing	
Van Woudenberg et al [65]	2-arm clustered RCT (10)	Promote PA	Smartphone-based SNI^i^ with MyMovez2 Wearable Lab—a smartphone with a tailor-made research app	Influential adolescents (school), Venlo, Netherlands	190 (male 88 and female 102)	12.7 (0.50)	11-19	SB (minutes per day) and MVPA (minutes per day); accelerometer (Fitbit Flex)	

^a^mHealth: mobile health.

^b^SB: sedentary behavior.

^c^IPA: insufficient physical activity.

^d^RCT: randomized controlled trial.

^e^PA: physical activity.

^f^MVPA: moderate to vigorous physical activity.

^g^PAQ-A: Physical Activity Questionnaire for Adolescents.

^h^IPAQ: International Physical Activity Questionnaire.

^i^SNI: social network intervention.

**Table 2:** Mobile health intervention characteristics: study aims, BCT^a^ cluster, theoretical foundation, and individualization.

Study (RCT^b^ and protocol) and intervention (study arm)		BCT taxonomy cluster, according to Michie et al [17] (N)	Theoretical foundation (N)	Individualization (N)	Level of individualization
**Chen et al [62]**					
	Fitbit app and Facebook	Goals and planning, feedback and monitoring, social support, shaping knowledge, comparison of behavior, reward and threat, and associations (7)	Not mentioned (0)	Competitions with community or friends, individual goal setting, task adjustment in relation to BMI, direct biofeedback and real-time coaching, goal-specific motivational coaching, personalized advice, and guidance (6)	High
**Nyström et al [61,66]**					
	MINISTOP app	Feedback and monitoring and associations (2)	Not mentioned (0)	Individual feedback (1)	Low
**Direito et al [58]**					
	Zombies, Run! app (1)	Goals and planning and feedback and monitoring (2)	Self-regulatory behavior change theory [67] (1)	Audio instructions, missions and defense bases, and web-based races (3)	Low
	Get Running app (2)	Goals and planning, feedback and monitoring, comparison of behavior, and reward and threat (4)	Self-regulatory behavior change theory [67] (1)	Human voice coach, training path, friend integration, low threshold approach, recovery periods, and music (6)	High
**Downing et al [63,68]**					
	Mini-Movers SMS text messaging–based intervention	Goals and planning, feedback and monitoring, and reward and threat (3)	Social cognitive theory [69], SMART^c^ goal framework [70], and CALO-RE^d^ taxonomy [71] (3)	Individual goal setting; goal-specific feedback; tailored SMS text messages; and just-in-time delivery of SMS text messages based on preferred time, date, and activity (4)	High
**Fassnacht et al [59]**					
	SMS text messaging–based feedback intervention	Goals and planning, feedback and monitoring, and associations (3)	Not mentioned (0)	Individual goal setting, task adjustment in relation to BMI, tailored feedback messages, and goal-specific motivational coaching (4)	High
**Gaudet et al [57]**					
	FitBit app immediate intervention (1)	Goals and planning, feedback and monitoring, social support, shaping knowledge, comparison of behavior, reward and threat, and associations (7)	Not mentioned (0)	Competitions with community or friends, individual goal setting, task adjustment in relation to BMI, direct biofeedback and real-time coaching, goal-specific motivational coaching, personalized advice, and guidance (6)	High
	FitBit app delayed intervention (2)	Goals and planning, feedback and monitoring, social support, shaping knowledge, comparison of behavior, reward and threat, and associations (7)	Not mentioned (0)	Competitions with community or friends, individual goal setting, task adjustment in relation to BMI, direct biofeedback and real-time coaching, goal-specific motivational coaching, personalized advice, and guidance (6)	High
**Hammersley et al [64,72]**					
	Time2b-Healthy Facebook and on the web	Goal setting, revision of goals, feedback, and challenges (4)	Self-efficacy model [73] and SMART goals framework [70] (2)	Tailored reminder emails, a Facebook group with individual goal setting, and goal-specific motivational coaching (4)	High
**Mendoza et al [60]**					
	Fitbit app and Facebook	Goals and planning, feedback and monitoring, social support, shaping knowledge, comparison of behavior, reward and threat, and associations (7)	Not mentioned (0)	Individual awards in a Facebook group, competitions with community or friends, individual goal setting, task adjustment in relation to BMI, direct biofeedback and real-time coaching, goal-specific motivational coaching, personalized advice, and guidance (7)	High
**Pyky et al [41,74,75]**					
	Clans of Oulu gamified app and web-based MOPO portal	Goals and planning, feedback and monitoring, social support, comparison of behavior, comparison of outcomes, reward and threat, associations, identity, and covert learning (9)	Transtheoretical Model of Behavior Change [76] (1)	Stage of behavior change, individual feedback on physical activity and sitting time, GPS-based tasks, competitions with community, and peer-referenced comparison (5)	High
**Woudenberg et al [65,77]**					
	App-based social network intervention—MyMovez	Comparison of behavior, reward and threat, and identity (3)	Theory of Planned Behavior [78], Self-Determination Theory [79], and Self-Persuasion Theory [80] (3)	Content tailored to influential youths, comparing individual scores with others, individual rewards, and individual identification with health behavior (4)	High
**Sirriyeh et al [56]**					
	Instrumental SMS text message intervention	Goals and planning, shaping knowledge, and identity (3)	Theory of Planned Behavior [78] (1)	Individual goal setting (1)	Low
	Affective SMS text message intervention	Goals and planning, self-belief, and identity (3)	Theory of Planned Behavior [78] (1)	Individual goal setting (1)	Low
	Combined SMS text message intervention	Goals and planning, shaping knowledge, self-belief, and identity (4)	Theory of Planned Behavior [78] (1)	Individual goal setting (1)	Low

^a^BCT: behavior change technique.

^b^RCT: randomized controlled trial.

^c^SMART: Specific, Measurable, Achievable, Relevant, and Time-Bound.

^d^CALO-RE: Coventry, Aberdeen, and London-Refined.

Among the 11 included studies, 3 (27%) had multiple study arms [56-58], resulting in a total of 15 mHealth interventions. In studies with multiple arms, each study arm represented a subintervention. Unfortunately, the subtrials of Sirriyeh et al [56] could not be integrated into the meta-analysis because of missing data. Overall, 33% (5/15) indicated a low level of individualization, and 66% (10/15) of interventions showed a high level of individualization. Individual goal setting was the most common technique used to individualize mHealth interventions. If the level of individualization in the studies was low, there was also a low use of BCTs in these interventions. The reporting of the theoretical foundation was not mentioned in 40% (6/15) of interventions and was therefore generally poor, although the interventions of Downing et al [68] and Woudenberg et al [65] were each based on 3 underlying theories. The most common theories were self-regulatory BCT [67]; Specific, Measurable, Achievable, Relevant, and Time-Bound goals framework [70]; Theory of Planned Behavior [78]; Self-Determination Theory [79]; Self-Persuasion Theory [80]; Transtheoretical Model of Behavior Change [76]; social cognitive theory [69]; and the Coventry, Aberdeen, and London-Refined taxonomy [71]. The number of behavior change elements correlated with the number of individualized elements. Of the 12 included interventions, 2 (17%) were SMS text messaging based, 5 (42%) included some form of social media (eg, Facebook), and 4 (33%) used the Fitbit app.

### ROB in Studies

Across the 11 studies, 7 out of 60 ratings (5 dimensions ×12 studies) indicated high ROB, and 7 ratings showed an unclear ROB, resulting in an overall rating of 3 (27%) studies with low, 2 (18%) studies with unclear, and 6 (55%) studies with a high ROB. Potential biases frequently occurred in dimensions A (bias arising from the randomization process) and D (bias in the measurement of the outcome). More detailed ROB information for each study can be found in Multimedia Appendix 1 [41,57-65] and Multimedia Appendix 2 and is also integrated into the forest plots for the meta-analysis.

### Synthesis of Results

#### Effects of High-Individualized and Low-Individualized mHealth Interventions on Decreasing IPA

Approximately 82% (9/11) of studies evaluated the effects of mHealth interventions on decreasing IPA levels, of which 22% (2/9) included multiple study arms [57,58]. Notably, the nonimmersive app of Direito et al [58] (arm 2) contributed to a reduction in IPA, whereas the immersive app (arm 1) increased IPA. One of the trials [56] was not included because of missing data on IPA. Splitted shared group procedure was used in studies with multiple study arms to avoid unit of analysis error [48]. As shown in Figure 2, the meta-analysis of IPA demonstrated a significant, small overall effect size (Cohen *d*=0.23; 95% CI 0.02-0.45; *Z*=2.13; *P*=.03). Trials with high levels of individualization (9/11, 82% of studies) significantly decreased IPA levels, with a moderate effect size (Cohen *d*=0.33; 95% CI 0.08-0.58; *Z*=2.55; *P*=.01). In contrast, those with low levels of individualization (2/11, 18% of studies) indicated no overall effect or even a nonsignificant increase in IPA (Cohen *d*=−0.06; 95% CI −0.32 to 0.20; *Z*=0.48; *P*=.63). A test for subgroup differences indicated that the described difference between interventions with high and low levels of individualization was statistically significant (*χ*^2^_1_=4.0; *P*=.04; *I*^2^=75.2%). The overall heterogeneity was moderate (τ^2^=0.02; *χ*^2^_9_=1.1; *P*=.002; *I*^2^=64%), and several ROB dimensions indicated a high ROB. As can be seen in Figure 2, dimensions A (bias arising from the randomization process), C (bias because of missing outcome date), and D (bias in the measurement of the outcome) were most frequently represented.

#### Effects of High-Individualized and Low-Individualized mHealth Interventions on Decreasing SB

Overall, all 10 included studies evaluated the effects of mHealth interventions on decreasing SB time, and 2 (20%) studies included multiple study arms [57,58]. The results showed a difference in positive effect sizes between the 2 arms of the Gaudet et al [57] study, although it was a crossover trial. In contrast, the Direito et al [58] immersive app (arm 1) showed a slight reduction in SB, whereas the nonimmersive app (arm 2) showed a slight increase. In contrast to the meta-analytic outcome measure IPA, the analysis indicated neither a significant subgroup difference between interventions with low and high levels of individualization (*χ*^2^_1_=0.4; *P*=.54; *I*^2^=0%) nor a general, significant effect within each subgroup (*Z*=1.70, *P*=.09; *Z*=.53, *P*=.59). Of the 15 interventions, 8 (53%) demonstrated a small increase in SB time. The heterogeneity of the included studies was overall low to moderate (τ^2^=0.01; *χ*^2^_11_=12.7; *P=*.31; *I*^2^=13%) but varied by subgroup (trials with high levels of individualization: τ^2^=0.02, *χ*^2^_9_=12.5, *P*=.19, *I*^2^=28%; trials with low level of individualization: τ^2^=0.00, *χ*^2^_1_=0.1, *P*=.70, *I*^2^=0%). As demonstrated in Figure 3, several ROB dimensions indicated an unclear or high ROB. Dimensions A (bias arising from the randomization process), C (bias because of missing outcome date), and D (bias in the outcome measurement) were the most frequently represented.

### Reporting Biases

Publication bias between studies was assessed using funnel plots for the 2 outcomes of IPA and SB. Statistical tests (eg, Egger regression [81]) for publication bias were not performed because of the small number of included studies.

**Figure 4:**
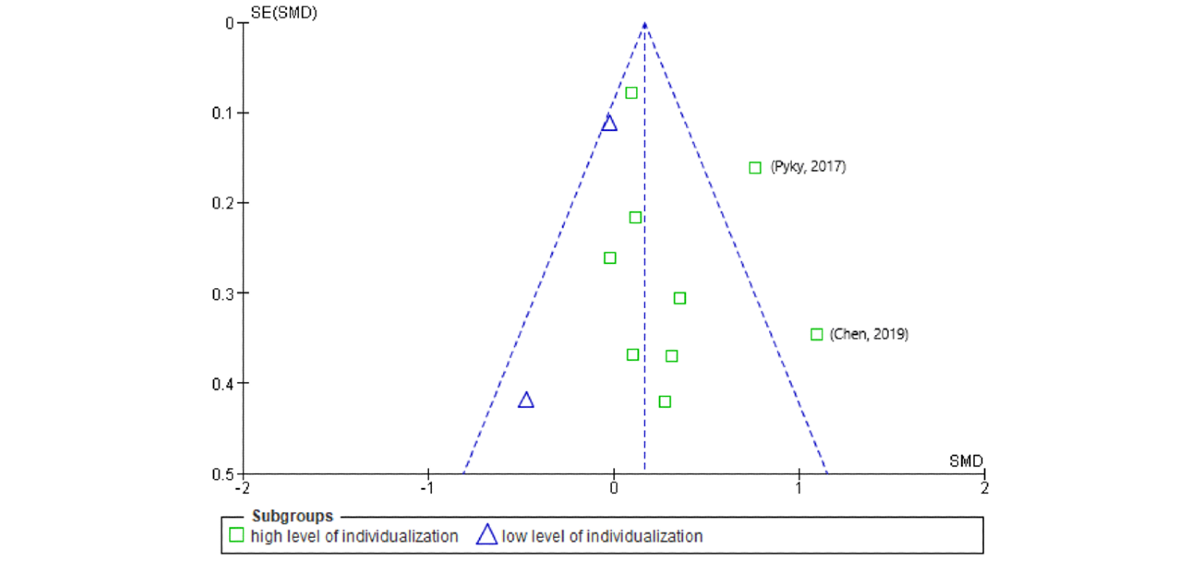
Funnel plot of comparison: insufficient physical activity outcomes. SMD: standardized mean difference.

**Figure 5:**
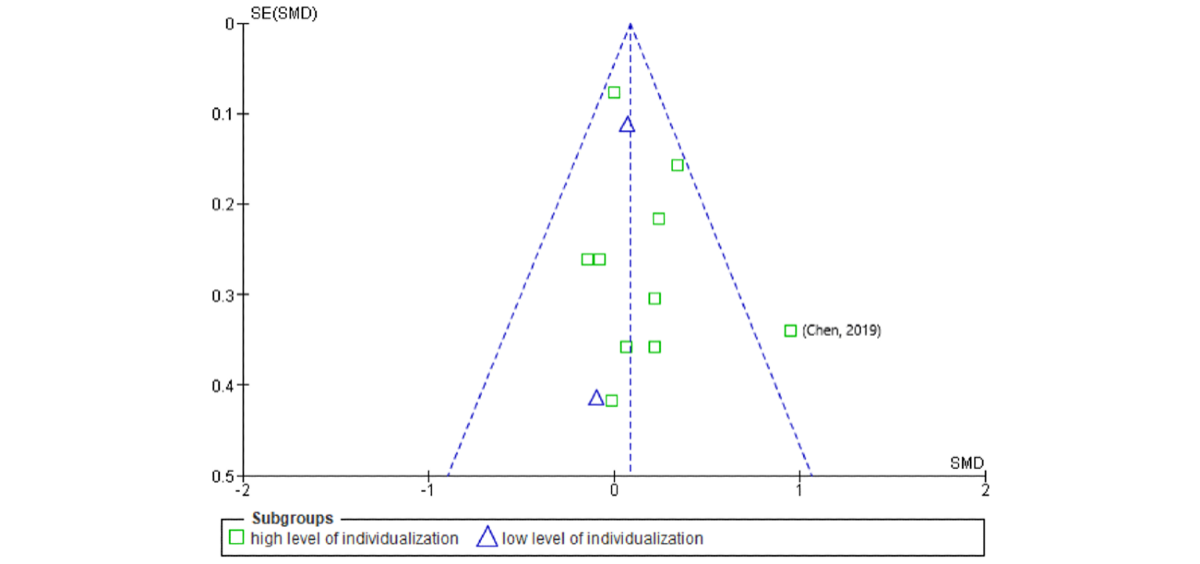
Funnel plot of comparison: sedentary behavior outcomes. SMD: standardized mean difference.

Visual inspection of funnel plots (Figures 4 and 5) indicated no serious publication bias in either case. The results of the study by Chen et al [62] occurred outside of the 95% CIs for both outcomes but for high-individualized trials only. Low-level individualization showed a smaller effect, and no results were outside the 95% CI. This also applies to the result of Pyky et al [41] for the IPA outcome. Therefore, it is particularly important to critically reflect on the results reported by Chen et al [62] and Pyky et al [41].

### Certainty of Evidence

As shown in Table 3, moderate confidence was evident in the meta-analysis effect estimate for IPA. The true effect is likely to be close to the estimate; however, there is a possibility that it is substantially different. By contrast, our confidence in the estimated effect is very limited for the primary outcome of SB, and the true effect may be substantially different. This potential bias is reinforced by the studies of Chen et al [62] and Pyky et al [41], which have above-average effect sizes while being severely weighted.

**Table 3:** Summary of findings based on Grading of Recommendations, Assessment, Development, and Evaluations approach (N=11).

Subgroup	Studies, n (%)	Study design	Risk of bias	Inconsistency	Indirectness	Imprecision	Publication bias	Relative risk (95% CI)	Certainty
IPA^a^, high level of individualization	7 (64)	RCT^b^	Not serious	Not serious	Not serious	Serious (−1)	Probably not	0.25 (0.02 to 0.47)	Moderate
IPA, low level of individualization	3 (27)	RCT	Not serious	Not serious	Not serious	Serious (−1)	Probably not	−0.05 (−0.24 to 0.15)	Moderate
SB^c^, high level of individualization	8 (73)	RCT	Not serious	Not serious	Not serious	Serious (−1)	Probably yes (−1)	0.12 (−0.07 to 0.32)	Low
SB, low level of individualization	4 (36)	RCT	Not serious	Serious (−1)	Not serious	Serious (−1)	Probably yes (−1)	0.74 (−1.08 to 2.55)	Very low

^a^IPA: insufficient physical activity.

^b^RCT: randomized controlled trial.

^c^SB: sedentary behavior.

### Additional Analyses

**Figure 6:**
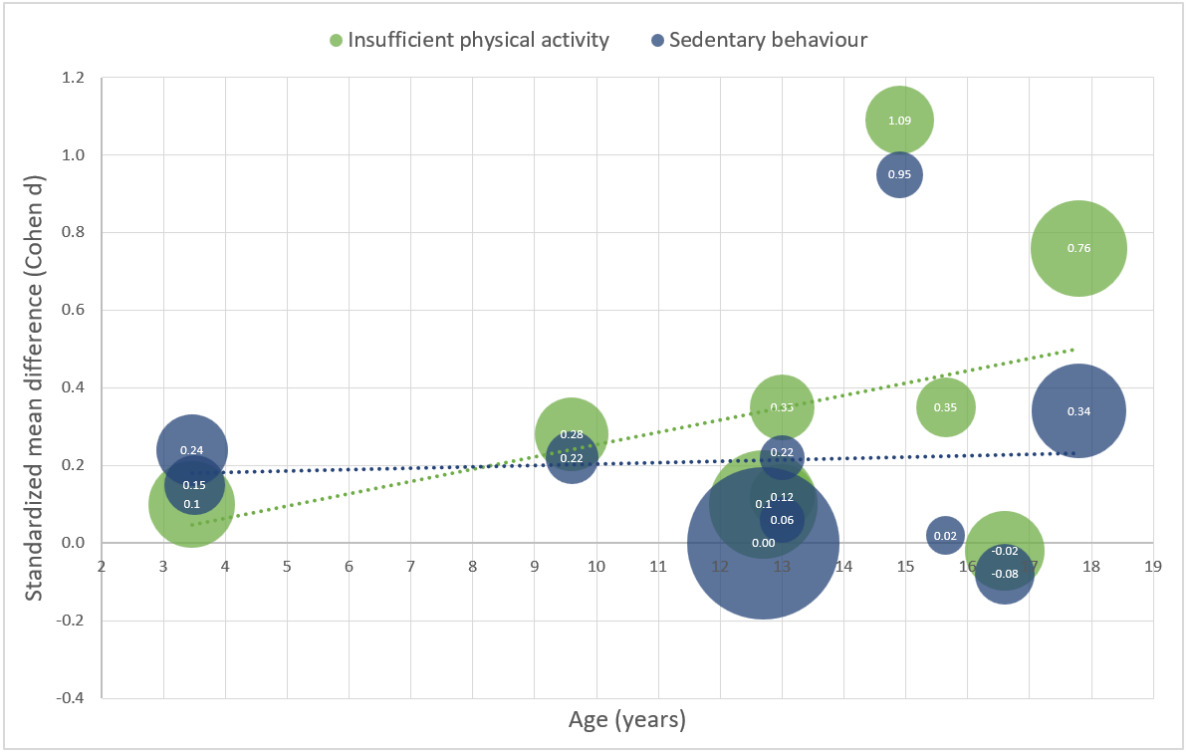
Grouped bubble plot of weighted standardized mean differences of individual trials and mean age of participants. Group differentiation based on the primary outcome (IPA and SB). High-individualized trials included only.

In an exploratory approach, the effect sizes obtained from the highly individualized interventions were further explored in a meta-regression analysis with age as a moderator variable to explain the moderate heterogeneity between studies and incorporate developmental psychological aspects of children and adolescents. Therefore, Figure 6 shows a weighted grouped scatter plot of the standardized mean differences (Cohen *d*) of individual interventions (including multiple study arms) and the mean age of participants. Group differentiation was based on the primary outcomes (IPA and SB). Meta-regression analysis results indicated that effect sizes were negligible for children (aged 1-14 years). There were nonsignificant differences in IPA in the adolescent age groups (14-18 years). Although the effect size (Cohen *d*) of highly individualized interventions with respect to SB remained approximately the same across age (τ^2^=0.0115, SE 0.0226; τ=0.1071; *I*^2^*=*21.23%; *H*^2^=1.72; *R*²=0.00%; test for residual heterogeneity: *QE*_10_=11.8472, *P*=.30; test of moderators: *QM*_1_=0.1451, *P*=.70) the effectiveness of highly individualized interventions of IPA increased slightly but not significantly across age (τ^2^=0.0564, SE 0.0546; τ=0.2375; *I*^2^*=*57.01%; *H*^2^=2.33; *R*²=28.47%; test for residual heterogeneity: *QE*_9_=20.3088, *P*=.02; test of moderators: *QM*_1_=2.0165, *P*=.16). Although the small number of included interventions allowed only descriptive conclusions to be drawn, the underlying tendency is evident in the data and needs to be examined in future studies.

## Discussion

### Principal Findings

This review and meta-analysis aimed to identify and characterize existing mHealth interventions for children and adolescents in the context of primary prevention of IPA and SB. In addition, this analysis aimed to provide clarity on whether and how effective mHealth interventions are in reducing IPA and SB in healthy children and adolescents. As a broad objective, we aimed to examine whether age and individualization influenced the overall effectiveness of mHealth interventions.

### Summary of Evidence

Out of 828 identified studies, a total of 11 (1.3%) were included for the qualitative synthesis and 10 (1.2%) for the meta-analysis based on the inclusion criteria. Trials included 1515 participants (mean age 11.69, SD 0.788 years; 65% male and 35% female) with self-reported (3/11, 27%) or device-measured (8/11, 73%) health data on the duration of SB and IPA for an average intervention period of 9.3 (SD 5.6) weeks (excluding follow-ups). Studies with high levels of individualization decreased IPA levels significantly (Cohen *d*=0.33; 95% CI 0.08-0.58; *Z*=2.55; *P*=.01), whereas those with low levels of individualization (Cohen *d*=−0.06; 95% CI −0.32 to 0.20; *Z*=0.48; *P*=.63) or addressing SB (Cohen *d*=−0.11; 95% CI −0.01 to 0.23; *Z*=1.73; *P*=.08) indicated no overall significant effect. Heterogeneity was moderate to low, and a test for subgroup differences indicated significant differences between trials with high and low levels of individualization (*χ*^2^_1_=4.0; *P*=.04; *I*^2^=75.2%). Age as a moderator variable showed a minor moderating effect; however, the results were not significant, which might have been because of being underpowered. This review is the first to examine the age- and individualization-dependent effectiveness of mHealth interventions to reduce IPA and SB in children and adolescents and strengthens the evidence of moderate mHealth effectiveness. This is in line with existing research on mHealth for children and adolescents [12,28].

### Characteristics of Observed mHealth Interventions

One of the main qualitative results concerning the first research question is that gamified approaches tend to have a higher effect in this population, and several previous interventions have already been shown to be effective [82,83]. The 18% (2/11) of trials showing the highest effectiveness in this meta-analysis (*Fitbit and Facebook intervention* by Chen et al [62] and the *Clans of Oulu intervention* by Pyky et al [41]) used this approach. However, it should be mentioned that the intervention *Zombies, Run!* by Direito et al [58], which showed a very low effect size, was also a gamified approach; however, it is hardly individualized and uses few BCTs. Therefore, the results suggest (in line with existing research [82]) that gamified approaches can be effective for children and adolescents but only if individualization, theoretical foundation, and integration of BCTs occur simultaneously. However, the 2 most effective interventions mentioned above are united by a distinguishing feature in addition to gamification. Both involve the social component and integrate community-based systems of social participation and association with real-world PAs in the surrounding environment. Hammersley et al [72] and van Woudenberg et al [65] integrated similar approaches. This may suggest that friends, family, and surrounding environments are relevant determinants for children and adolescents in the context of mHealth and should be considered in the development of mHealth interventions to reduce inadequate PA and SB.

This review also demonstrates that mHealth interventions for children and adolescents are rarely theory based [18,24,25], although theories were occasionally mentioned, and therefore reinforce the need for enhanced theoretical substantiation in the development of mHealth interventions. The consequences of non–theory-based approaches include low effect sizes and methodological deficiencies, at least in self-developed interventions [59,61]. No negative effect of missing theoreticity could be shown when already existing and evaluated apps (eg, Fitbit app) were used [57,60]. In this respect, another striking aspect of the results is that most of the considered interventions used commercially available apps (especially Fitbit models and the corresponding app) or self-developed approaches. Models from other well-known commercial providers were not used. Data transfer software was often cited as a reason in some studies. From a scientific point of view, one of the problems may be that Fitbit does not disclose the mechanisms and underlying theories behind its development.

Regarding the quality of the integrated data, it should be mentioned that many trials addressed multiple outcomes [84] and used questionnaire data as outcome parameters [85]. A more appropriate approach would be to focus only on objective data or consider a combination of objective and subjective data, similar to the approach of Chen et al [62]. The use of only qualitative data can become a problem if an objective comparison with WHO recommendations has to be provided [86]. Therefore, we encourage researchers in the field of mHealth to use accelerometry-based measurements and more standardized outcome measures in future intervention studies.

Another key aspect of qualitative analysis is the individualization of the included mHealth interventions. It is noticeable that the type of individualization varies considerably between techniques that are frequently used (eg, *individual goal setting*) and other techniques that are unique to one of the interventions (eg, individualization based on the stage of behavior change). Similar to existing ideas in the field of behavior change mechanisms [17], a consistent taxonomy is needed and should be a part of future research.

### Effectiveness of Observed mHealth Interventions

Across all interventions, it appears that mHealth interventions to reduce IPA in children and adolescents showed an overall significant moderate effectiveness, whereas interventions to reduce SB showed no overall significant effect. Accordingly, it appears easier to change IPA than SB in children and adolescents. More structural changes are probably necessary to reduce SB, which include educational policies for schools. For instance, it is harder to reduce sitting time in class, at lunch, at home while doing homework, or during transportation than it is to do another hour of sports in the evening. Potential ideas that could be implemented in the context of mHealth would be *just-in-time adaptive interventions* with reminders for small exercise breaks [20]; in the school context, the use of automated standing desks to interrupt sitting times; or the assignment of physically activating homework that encourages children and adolescents to explore their invigorated environment.

It should be further discussed that the considered mHealth interventions had no or even a small reverse effect on the reduction of SB. Although it has been shown that screen time and PA are independent constructs [33,34], it becomes evident that the use of apps leads to as much or slightly more time spent in SB, although IPA decreases. Thus, there is presumably a shift in time resources among children and adolescents through the use of mHealth intervention. A similar finding emerged for the game Pokémon Go [82]. The consequences of this finding are far-reaching and suggest that the use of mHealth in adolescence and childhood deserves careful consideration. For younger age groups, in particular, the use of an app as a family or with parental support could make sense but results in low effect sizes, as shown by 20% (3/15) of the considered interventions [61,64,68].

### Moderating Effects of Individualization and Age

Looking at the average age of the target groups in the interventions used in the meta-regression, it is noteworthy that the highest effect sizes were evident in adolescent age groups. Therefore, it is reasonable to assume that participants in different age groups are differently impressionable by mHealth. There are multiple explanations for this finding. First, as children age, unhealthy behaviors may be established, and apps may need to become more individualized to be effective [21]. Second, the more the child evolves into an individual, the more important it becomes to address their individuality in health interventions. The second hypothesis is supported by one of the key findings of the meta-analysis that individualized mHealth interventions to reduce IPA differ significantly from nonindividualized interventions with the same objective. This is in line with previous research on other populations [21]. However, it is interesting to note that interventions with the most individualized elements are not the most effective [60]. Thus, more individualization does not necessarily lead to higher effectiveness; rather, the selection of particular relevant parameters in combination with the rest of the intervention characteristics seems to result in an effective intervention. For example, the development of a new intervention could be accompanied by a kind of intervention mapping [87] accompanied by a target group analysis. This would reveal the needs and requirements of the target group of an mHealth intervention. Future research should aim to deepen these partially exploratory findings and identify the underlying psychological mechanisms. We hypothesize that there is a sweet spot at which the addition of further mechanisms for individualization and behavior change no longer leads to a larger effect, which would have severe implications for the development of mHealth interventions. Furthermore, based on the results of this review, we would like to point out that the content and functions of mHealth interventions for children and adolescents should always be adapted to the age of the target group to avoid possible developmental psychological difficulties and associated low effect sizes. It should also be mentioned that the results of the meta-regression, as suggested in the Introduction section, again indicate that SB and IPA are not correlated constructs. Therefore, PA promotion does not necessarily imply SB reduction. Therefore, mHealth should be addressed separately.

### Strengths and Limitations

This review is the first to differentiate between SB and IPA when considering the effects of mHealth on children and adolescents and contrast both study effects and bias. Moreover, no other review in the field to date includes a narrative analysis of individualized elements in mHealth interventions and relates them to intervention effectiveness. Another unique feature is the exploratory meta-regression. In addition to these strengths, this review has numerous limitations, both at the study and review levels.

At the study level, apart from the studies by von Pyky et al [41], van Woudenberg et al [65], and Nyström et al [61], the sample size was generally moderate to small, which may have biased the results. It should also be noted that most of the studies included multiple outcome parameters and that the primary objective of these studies was not to decrease IPA and SB. As a consequence, we assume that the observed effect sizes do not fully reflect the magnitude of the true effect. If all the included mHealth interventions were targeted at reducing IPA or SB alone, the results would certainly be more conclusive. Conspicuous among studies with small sample sizes compared with those with larger samples is the lower rating in the ROB assessment. In addition, there was a small number of included studies and partly considerable heterogeneity because of deviants, for example, the results of the study by Pyky et al [41]. This could be because of the major variability in the study design or the diverse target and age groups.

At the review level, the asymmetries observed in the funnel plot of the SB outcome indicate a publication bias. This is probably because of the study by Pyky et al [41], although the ROB assessment in this study was positive. Furthermore, it should be noted that the study results of Sirriyeh et al [56] could not be included in the meta-analysis because of a lack of reporting and as the authors did not provide any data when asked repeatedly. As the study was a 4-arm randomized controlled trial, this would certainly have been insightful for the review. In the included studies with several study arms, such as that of Direito et al [58], it was observed that the results of individual studies sometimes differed considerably. In this case, the immersive app *Zombies, Run* showed a substantially smaller effect than the nonimmersive app *Get Running*. Although other existing meta-analyses in the field of mHealth for children and adolescents similarly integrate multiple study arms (eg, He et al [29]) and we attempted to avoid potential overpowering by using the splitted shared group procedure [48], this approach should be considered controversial. Arguably, 1 author team was responsible for an excessive degree of evidence. For example, if a study shows a high ROB and includes 4 study arms, it leads to a globally insufficient certainty of evidence. As the only way to avoid this potential bias is to deliberately exclude existing evidence, further research should focus on minimizing the number of study arms and developing new statistical methods to address this issue. Another limitation of this review was that follow-up data were not extracted. As mHealth in children and adolescents is still a relatively young field of research, we did not consider there to be enough studies with follow-up measurements for a meta-analysis and therefore decided not to include follow-up measurements for reasons of evidence comparability. However, concerning mHealth in adults, it has already been shown that the effects of the interventions decrease in the long term [13]. If more mHealth trials with children and adolescents become published, we suggest replicating this review, including its follow-up effects. We assume that the long-term effects are considerably stronger in children and adolescents than in adults, as they may not yet be as well-established as for adults.

In general, the results of this review and meta-analysis should be interpreted with caution, as only moderate to low certainty of evidence is warranted based on the Grading of Recommendations, Assessment, Development, and Evaluations rating. In addition, many publications identified in the systematic literature screening were excluded as they were study protocols or small pilot studies. Therefore, this review should be updated at a later date. Furthermore, there is also limited comparability between the included studies, as the mechanisms of the considered mHealth interventions certainly move along disparate causal pathways in different age groups.

### Conclusions

The findings of this review suggest that the considered mHealth interventions for healthy children and adolescents can foster low to moderate reductions in IPA but not SB. As no significant effects were shown for SB, future studies should identify how targeted SB can be reduced using mHealth. In the future, it may also be useful to test the described interventions in clinical populations (eg, children and adolescents diagnosed with obesity or metabolic syndrome), as distressing pressure may be greater here, potentially increasing adherence to use. Moreover, individualized mHealth interventions to reduce IPA are more effective for adolescents than for children. Although only a few mHealth studies have addressed inactive and sedentary young people, and their quality of evidence is moderate, these findings indicate the relevance of individualization in the period of adolescence on the one hand and the difficulties in reducing SB with mHealth interventions on the other. Future research and policy makers should aim to strengthen the evidence and systematically evaluate individualized mHealth interventions for children and adolescents. Especially in multidisciplinary collaborations among app development, science, and engineering, there is great potential for high-quality mHealth intervention development.

## Appendix

Multimedia Appendix 1 Risk of bias in individual studies.

Multimedia Appendix 2 Risk of bias across studies.

## Abbreviations

BCT: behavior change technique
IPA: insufficient physical activity
MET: metabolic equivalent of task
mHealth: mobile health
MVPA: moderate to vigorous physical activity
PA: physical activity
PRISMA: Preferred Reporting Items for Systematic Reviews and Meta-Analyses
PROSPERO: International Prospective Register of Systematic Reviews
ROB: risk of bias
SB: sedentary behavior
WHO: World Health Organization
