# Supplementary figures and images for: mHealth Interventions to Reduce Physical Inactivity and Sedentary Behavior in Children and Adolescents: Systematic Review and Meta-analysis of Randomized Controlled Trials
Source: JMIR Mhealth Uhealth. 2022 May 11;10(5):e35920. doi: 10.2196/35920 (PMC9133983; doi:10.2196/35920)

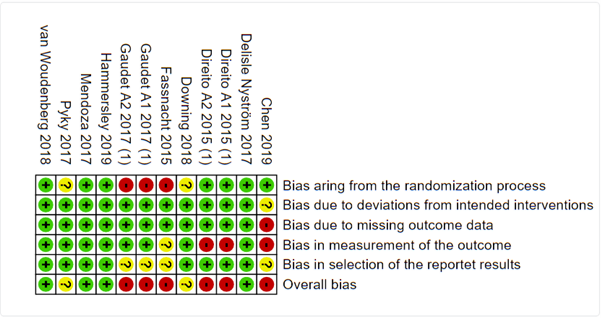

Supplement: Multimedia Appendix 1 [file mhealth_v10i5e35920_app1.png]

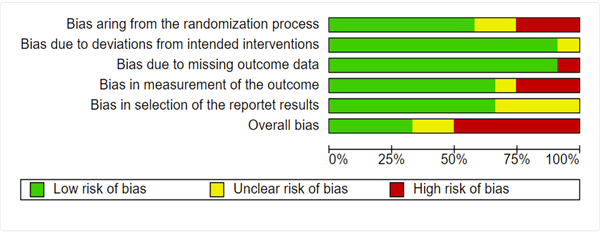

Supplement: Multimedia Appendix 2 [file mhealth_v10i5e35920_app2.png]
